# Exciting science from the 16th International Conference on Thalassemia and Hemoglobinopathies

**DOI:** 10.1002/hem3.114

**Published:** 2024-07-19

**Authors:** Dimitrios Farmakis, Michael Angastiniotis, Androulla Eleftheriou

**Affiliations:** ^1^ Thalassaemia International Federation Nicosia Cyprus; ^2^ National and Kapodistrian University of Athens Medical School Athens Greece

The 16th International Conference on Thalassemia & Hemoglobinopathies was held together with the 18th International Conference for Patients and Parents in Kuala Lumpur, Malaysia, on November 3–5, 2023. Both congresses were organized by the Thalassaemia International Federation (TIF), a patient‐oriented, nonprofit, nongovernmental umbrella federation with 231 member associations from 69 countries. TIF works in official relations with the World Health Organization (WHO), the European Council, and the United Nations Economic and Social Council to promote access to quality care for all patients with thalassemia or other hemoglobinopathies worldwide.

The scientific program of the conference addressed a broad range of topics concerning thalassemia, sickle cell disease (SCD), and other rare anemias, providing a comprehensive review of the current state of the art, recent advances, and persisting or emerging challenges on diagnosis, management, and prevention of hemoglobinopathies.

## Therapeutic advances

Impaired maturation of erythroid progenitors to red cells in bone marrow is a key component of β‐thalassemia pathophysiology. Luspatercept is a novel drug that promotes erythroid maturation, improving anemia and reducing transfusional requirements. Its efficacy and safety have been documented in transfusion‐dependent thalassemia (TDT) and nontransfusion‐dependent thalassemia (NTDT) by the randomized trials BELIEVE and BEYOND, respectively,^1^, ^2^ resulting in the approval of the drug for TDT in 2020 and for either TDT or NTDT in 2023. Open issues include the efficacy and safety of luspatercept in children, patients with alpha‐thalassemia, and in combination with other drugs and the identification of predictors of response and side effects. The high cost of the drug poses a challenge to healthcare systems and creates the need for proper patient selection.

Mitapivat is an oral small‐molecule activator of red cell‐specific pyruvate kinase (PK). PK is crucial for the energetic supply, function, and survival of red cells, which are compromised in patients with PK deficiency, thalassemia, and SCD. A series of clinical trials have addressed the efficacy and safety of mitapivat in these three hemolytic conditions. Two phase 2 studies in NTDT and SCD, respectively, documented improvements in hemoglobin levels, hemolysis, and sickling, with adequate safety.^3^, ^4^ Two phase 3 trials in TDT and NTDT and a larger phase 2/3 study in SCD are ongoing.

Drugs and interventions promoting hemoglobin F (HbF) synthesis have been tested as potential therapies for β‐thalassemia. BCL11A is a transcription factor inhibiting HbF synthesis and its genetic manipulation with the gene‐editing technique CRISPR‐Cas9, followed by autologous stem cell transplantation with BCL11A‐edited cells, is being evaluated in β‐thalassemia and SCD.^5^

The TMPRSS6 antisense oligonucleotide (ASO) inhibits the expression of TMPRSS6, a regulator of hepcidin, resulting in its increased synthesis. The promotion of hepcidin synthesis would prevent iron overload, and might also improve anemia, particularly when combined with an erythroid maturation agent. Preliminary data show that TMPRSS6‐ASO and luspatercept combination induces improvement in anemia, iron overload, ineffective erythropoiesis, and splenomegaly, an approach that remains to be confirmed by clinical trials.^6^

Crizanlizumab, a P‐selectin antibody aiming at preventing vaso‐occlusion, has shown benefit in a phase 3 study, but follow‐up data remain unclear, while its future development is under review.^7^ Voxelotor, a hemoglobin S polymerization inhibitor, has shown a beneficial effect on hemoglobin levels but its effect on vaso‐occlusive crises is not yet proven.^7^

Gene therapy offers an alternative curative treatment approach that overcomes the limitations of hematopoietic stem cell transplantation.^8^ Regarding gene addition, pooled data from clinical studies with the beti‐cel regimen have overall shown transfusion independence in 82% of patients. However, the considerably high cost of this therapy limits substantially patients' access.

## Persisting challenges

Thalassemia poses a huge public health burden in high‐prevalence countries and prevention remains the best approach to address it. However, thalassemia prevention and management continue to face several challenges in developing countries, including poverty, lack of epidemiological data, lack of awareness, high prevalence of communicable diseases, and a series of ethical, social, religious, and legal issues. More than 300,000 babies affected by hemoglobinopathy are born annually, while only 12% of TDT patients globally receive adequate transfusion therapy and less than 40% of those transfused receive adequate iron chelation. On the other hand, the continuously increasing costs of treatment render the continuity of care delivery a major challenge for healthcare systems. In the United Kingdom, the lifetime cost of treatment to 50 years of age is GBP 483,454 and this cost has increased by 32% in the last 16 years.^9^

The safety of blood products can be guaranteed by proper regulatory frameworks, low‐risk volunteer nonremunerated blood donors, and high‐quality, robust evidence‐based processes in collection, testing, preparation, and storage. However, WHO data show a marked heterogeneity across regions and countries in the proportion of voluntary nonremunerated blood donation or the presence of haemovigilance systems or national transfusion policies and regulations.

“*Transition*” is a planned process that supports adolescents and young adults with chronic health conditions to move from child‐centered to adult‐oriented healthcare providers and facilities. Up to 70% of adults with thalassemia are receiving care in pediatric centers and less than 40% participate in dedicated transition programs.

Advances in thalassemia management over the past decades have resulted in a dramatic improvement in survival rates, with the majority of patients in populations with access to proper care surviving until the age of 50. This aging patient population is expected to develop new forms of age‐related complications such as hepatocellular carcinoma and other malignancies, atrial fibrillation, aortic stenosis, heart failure with preserved left ventricular ejection fraction, bone disease, renal disease, chronic pain, and depression, that would, in turn, create new needs for monitoring and management.^10^

## TIF activities update

In 2023, TIF published three new guideline documents that were officially presented at the 16th Conference, including the first guidelines for the management of α‐thalassemia, an updated edition of the guidelines for the management of NTD β‐thalassemia and the first guidelines for clinicians on nutrition for thalassemia and SCD. All documents are accessible through TIF's website (https://thalassaemia.org.cy/).

Education is one of the main pillars of TIF's work. Educational resources for healthcare professionals include conferences and workshops, fellowships and preceptorships, publications as well as electronic and online resources such as the TIF e‐Academy, offering online self‐paced courses, and the Thalassaemia International Federation Library eXtended, an extensive library of video recordings from webinars, workshops and conferences presented by renowned international experts. Similar resources are available for patients, also including the THALIA App, developed under the HORIZON 2020 THALIA project, designed to help patients self‐manage their disease on a daily basis. All resources are available completely free of charge through TIF's website (https://thalassaemia.org.cy/).

## AUTHOR CONTRIBUTIONS

**Dimitrios Farmakis**: Conception and drafting. **Michael Angastiniotis**: Conception and critical review. **Androulla Eleftheriou**: Conception and critical review.

## CONFLICT OF INTEREST STATEMENT

The authors declare no conflict of interest.

## FUNDING

This research received no funding.

## Data Availability

Data sharing is not applicable to this article as no new data were created or analyzed in this study.
